# The association of systemic immune-inflammation index with lung function, risk of COPD and COPD severity: A population-based study

**DOI:** 10.1371/journal.pone.0303286

**Published:** 2024-06-14

**Authors:** Ying-da Song, Xiao-ming Bai, Jun Ma

**Affiliations:** 1 Department of Thoracic Surgery, Shanxi Provincial People’s Hospital, Taiyuan, Shanxi Province, China; 2 Fifth Clinical Medical College, Shanxi Medical University, Taiyuan, Shanxi Province, People’s Republic of China; National and Kapodistrian University of Athens, GREECE

## Abstract

**Purpose:**

The relationship between the levels of Systemic Immune-inflammation Index (SII) and chronic obstructive pulmonary disease (COPD), lung function, and COPD severity were not fully understood. We conducted this cross-sectional, population-based study to investigate the complex association between SII and COPD, lung function, and COPD severity among the US adults.

**Methods:**

Overall, 18,349 participants were included in the National Health and Nutrition Examination Survey (NHANES) between 2005 and 2018. The exposure variable was SII, calculated from platelet counts, neutrophil counts, and lymphocyte counts. Weighted univariable and multivariable logistic regression, subgroup analysis, and restricted cubic spline (RCS) regression were performed to assess the relationship between COPD, lung function, COPD severity and SII. Last, we used a propensity score matching (PSM) analysis to reduce selective bias and validate these relationships.

**Results:**

Approximately 1,094 (5.96%) of the participants were diagnosed as COPD. The multivariable-adjusted odds ratio (OR) (95% confidence interval, CI) for the Q2 group (Log-SII > 2.740) was 1.39 (1.16 to 1.68). Before and after matching, multivariable logistic regression models revealed that increased Log-SII levels (SII Logarithmic transformation) associated positively with the risk of COPD. The subgroup analysis showed no interaction between Log-SII and a variety of variables (P for interaction > 0.05). RCS showed a reversed L-shaped relationship between Log-SII with COPD (P for nonlinear = 0.001) in individuals. In addition, we observed negative significant correlations between forced expiratory volume in one second (FEV1) / forced vital capacity (FVC) %, FEV1/FVC% predicted and SII, and reversed U-shaped curve relationships between FEV1, FEV1% predicted and SII. High SII level is associated with severity of COPD, especially at Global Initiative on Obstructive Lung Disease (GOLD) 1 and GOLD 3.

**Conclusions:**

In summary, the Log-SII level is associated with COPD risk, lung function, and COPD severity.

## Introduction

Chronic Obstructive Pulmonary Disease (COPD) is a heterogeneous lung condition characterized by chronic respiratory symptoms due to abnormalities of the airways and/or alveoli that cause persistent, often progressive, airflow obstruction [1]. COPD is characterized by irreversible restriction of airflow and infiltration of chronic inflammatory cells such as macrophages, neutrophils, and T lymphocytes [2, 3]. In addition, COPD is characterized by high morbidity, disability rate, and mortality rate. Since the 20th century, with the transformation of economic models, genetic aspects, exposure to danger, and the improvement of residents’ living standards, COPD has become the third leading cause of death worldwide [4]. These will have a serious impact on human mental and physical well-being, as well as adding considerable economic pressure on families and society. It is estimated that the global economic cost of COPD will rise to 480 million dollars by 2030 [5]. Therefore, early identification and intervention are essential for COPD patients in order to slow the progression of the disease and improve the quality of life.

The Systemic Immune-inflammation Index (SII) is a reliable measure of inflammation and immune status based on the count of neutrophils, lymphocytes, and platelets [6]. The SII has become a popular research topic in the diagnosis and prognosis of various diseases. As in many previous studies, the SII is an important tool for predicting the prognosis and survival rate of patients with malignant tumors, spontaneous cerebral hemorrhage, and cerebral infarction. This index could provide an accurate assessment of a patient’s inflammatory and immune status, allowing clinicians to gain a better understanding of the patient’s condition and develop an appropriate treatment plan [7–11].

COPD is a complex pathophysiological disorder characterized by systemic inflammation, immune dysfunction, and airflow restriction. Herein, we hypothesized that SII is positively associated with COPD risk and severity of COPD, and SII is negatively associated with lung function. To better understand this relationship, we conducted a study utilizing data from the National Health and Nutrition Examination Survey (NHANES) from 2005 to 2018.

## Method

### Source of data and study population

The NHANES is a national cross-sectional study assessing the health and nutrition status in the United States. Relevant interview, examination, dietary, and laboratory data were collected from adults and children of the United States population through the method of “stratified multistage probability sampling”. The US National Center for Health Statistics (NCHS) Research Ethics Review Board approved all studies of the NHANES protocol, and each participant signed written informed consent. All data from the NHANES database have undergone ethical approval statement that approved by the National center for health statistics Ethics Review board approval, for details please see the NHANES website (https://www.cdc.gov/nchs/.nhanes/index.htm).

**Fig 1:**
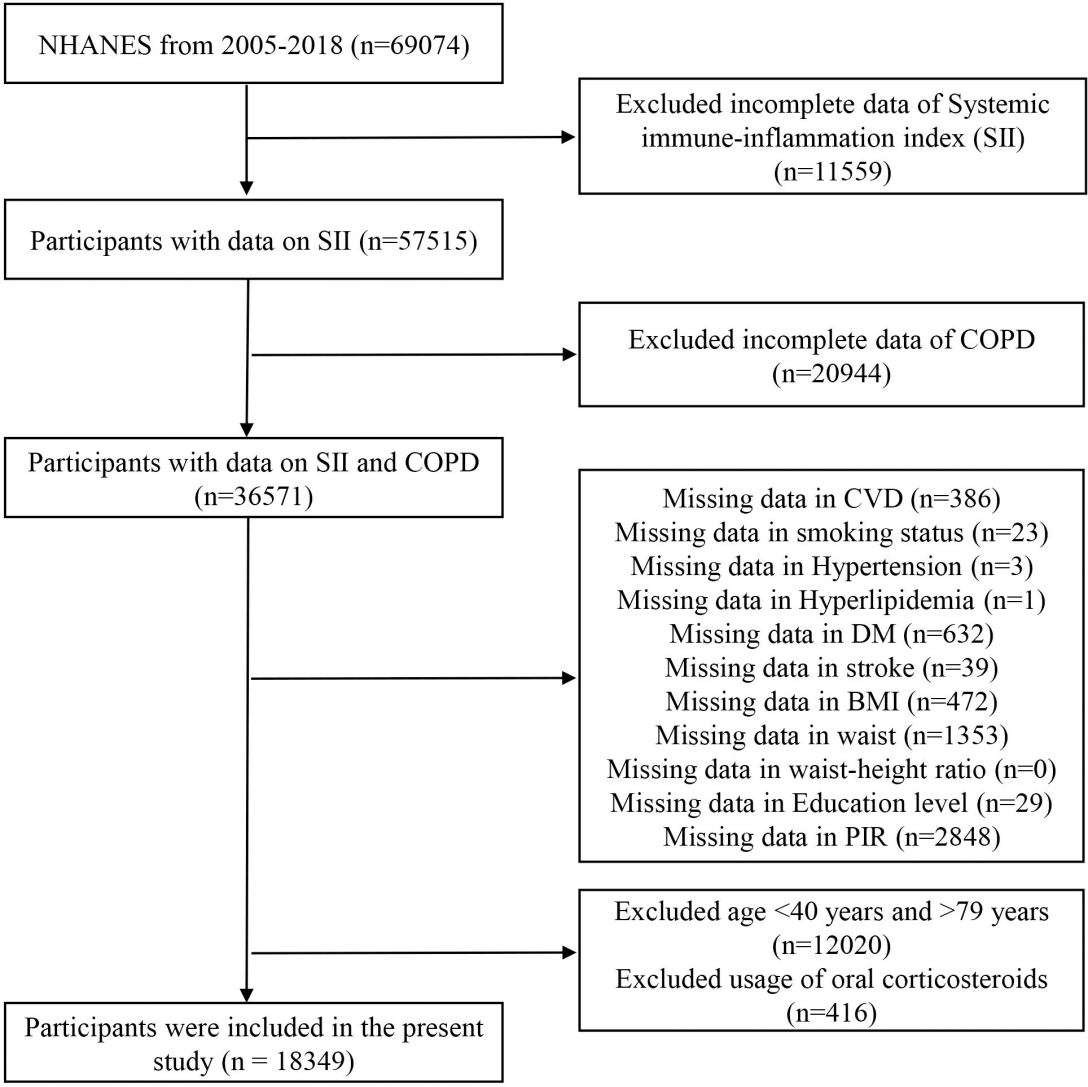
Flow diagram.

In our study of SII and COPD, a total of 18,349 participants were chosen from cohorts of 2005–2018 consecutive NHANES cycles. Exclusion criteria were as follows: age of participants was < 40 years or ≥ 80 years; participants without SII data; participants without COPD data; covariates with missing values; usage of oral corticosteroids in participants (Fig 1). Pulmonary function data exists only in the NHANES database 2007–2012. Thus, we selected the data of these years and conducted a separate analysis. The exclusion criteria were consistent with the above in our study of pulmonary function, severity of COPD and SII.

### Calculation of systemic immune-inflammation index

The development and calculation of the SII have been reported previously [6]. The SII is composed of platelet count, neutrophil count, and lymphocyte count (expressed as ×10^3^ cells/μL), that are collected from serum samples of participants and were measured with Coulter DxH 800 analyzer in the Collaborative Laboratory Services, Ottumwa, Iowa. The computed formula of the SII is as follows: platelet count× neutrophil count/lymphocyte count.

### Evaluation of chronic obstructive pulmonary disease

The diagnostic criteria for COPD were as follows: (1) FEV1/FVC < 0.7 in post-bronchodilator; (2) use drugs of selective phosphodiesterase-4 inhibitors, mast cell stabilizers, leukotriene modifiers or inhaled corticosteroids, age ≥ 40, and have smoke history or chronic bronchitis; (3) questionnaire surveys of participants including “ever told you had emphysema (mcq160g)?” and “ever told you had COPD, emphysema, chronic bronchitis (mcq160p)?”.

### Covariates

Selection of covariates that could be potential confounders of the associations between SII and COPD was based on the existent literature and clinical consideration. The standardized household interviews were used to obtain the demographic characteristics, including age, sex, race, educational level, and poverty index ratio (PIR). Age was divided into two age groups (40–59 years, and 60–79 years), with the 40–59 years as the reference; Race was divided into non-Hispanic white, non-Hispanic black, Mexican American, and others, with the non-Hispanic white as the reference; Education level was graded into primary school and less, middle and high school, and college and higher, with the college and higher as the reference. Poverty was defined as PIR ≤ 1.0, and divided into two categories of PIR (≤ 1.0 and > 1.0), with the PIR ≤ 1.0 as the reference. White blood cells (Wbc) count, platelets (Plt) count, neutrophils (Neu) count, lymphocytes (Lym) count, hemoglobin (Hb) level, Eosinophils (Eos) count and Monocyte (Mono) count were obtained from the laboratory data. Body mass index (BMI), waist-height ratio and waist circumference were obtained from the examination data. Diabetes (DM), cardiovascular disease (CVD), hypertension, hyperlipidemia, stroke, asthma and smoking status are potentially important risk factors for COPD. Therefore, these diseases were included in the analysis. The diagnostic criteria for DM were: (1) diagnosed with diabetes by doctors; (2) glycohemoglobin HbA1c > 6.5%; (3) fasting glucose ≥ 7.0mmol/l;(4) random blood glucose ≥ 11.1mmol/l;(5) two-hour Oral Glucose Tolerance Test (OGTT) blood glucose ≥ 11.1mmol/l;(6) use of diabetes medication or insulin. CVD was an aggregate of congestive heart failure, coronary heart disease, heart attack, angina, and stroke. The diagnostic criteria for hypertension were: (1) average systolic blood pressure ≥ 140 mmHg or average diastolic blood pressure ≥ 90 mmHg after at least three times of measurement; (2) use of anti-hypertensive drugs; (3) subject or physician-reported hypertension diagnosis. The diagnostic criteria for hyperlipidemia were: (1) total cholesterol level ≥ 5.18 mmol/L; (2) triglyceride level ≥ 150 mg/dL; (3) high-density lipoprotein-cholesterol < 1.04 mmol/L in males and 1.30 mmol/L in females; (4) low-density lipoprotein-cholesterol ≥ 3.37 mmol/L; (5) use of anti-hyperlipidemic drugs. The diagnostic criteria for stroke were based on questionnaire surveys of “ever told you had stroke (mcq160f or spq070d)?” The diagnostic criteria for asthma were: (1) use antiasthmatic drug; (2) use drugs of selective phosphodiesterase-4 inhibitors, mast cell stabilizers, leukotriene modifiers, or inhaled corticosteroids, age < 40, and have no smoke, chronic bronchitis and emphysema; (3) questionnaire surveys of participants including “ever told you had asthma (mcq010)?”. Smoking status was defined into three categories: (1) never: smoked less than 100 cigarettes in life; (2) former: smoked more than 100 cigarettes in life and smoke not at all now; (3) now: smoked moth than 100 cigarettes in life and smokes some days or every day. No or never was taken as the reference for all of the above diseases described.

### Lung function measurements and COPD severity

In the NHANES respiratory health spirometry procedures manual, the 1st test spirometry data represents pre-bronchodilator spirometric measurements, the 2nd test spirometry data are post-bronchodilator spirometric measurements for participants whose forced expiratory volume in one second (FEV1) / forced vital capacity (FVC) % was below the lower limit of normal values (LLN) determined according to their age, sex, weight, height and race/ethnicity, or whose FEV1/FVC% was below 70% in the 1st test spirometry data. Lung function standard measurements are assessed according to the recommendations of American Thoracic Society (ATS) [12]. We used the quality level of A (Exceeds ATS data collection standards) and B (Meets ATS data collection standards) for subsequent analysis. The severity of COPD is classified according to the Global Initiative on Obstructive Lung Disease (GOLD) staging system. GLOD 1 is post-bronchodilator FEV1/FVC% of less than 0.7 and FEV1 ≥ 80% predicted; GLOD2 is post-bronchodilator FEV1/FVC% of less than 0.7 and FEV1 between 50 and 80% of predicted; GLOD3 is post-bronchodilator FEV1/FVC% of less than 0.7 and FEV1 between 30 and 50% of predicted.

In this study, we focused on three pulmonary function indices, including FEV1, FVC, and FEV1/FVC. LLN and predicted % for three indicators were calculated based on sex, race/ethnicity, height and age according to the Global Pulmonary Function Initiative 2012 equation [13], NHANES III equations [14] and Hankinson et al. [15]. The 1st test spirometry data were used to study the association between SII and lung function, the 2nd test spirometry data were used to study the association between SII and severity of COPD.

### Statistical analysis

Considering the complexity of the sampling survey, each study subject was a weighted statistical analysis. Continuous variables were presented as mean (standard deviation (SD)), categorical variables were summarized as frequency (percentage). For baseline characteristics, categorical variables were compared using the chi-square test, and continuous variables were compared using the t-test or one-way analysis of variance. Moreover, for the non-normal distribution of SII data, we used Log transformations to obtain normal distributions of data (named Log-SII) by validation of Shapiro-Wilks tests. Receiver operating characteristic (ROC) curves were used to calculate cut-off values and evaluate sensitivity and specificity for different Log-SII in predicting COPD. A univariable logistic regression analysis was conducted to assess for risk factors of COPD. Multivariable logistic regression models (crude models to model 3) were used to investigate the relationship between Log-SII and COPD after progressively adjusting for different potential confounders. The crude model was not adjusted any covariates. Model 1 was adjusted for age, PIR, race, smoking status, and education. Model 2 was further adjusted for Wbc count, Mono count, Eos count, waist-height ratio and waist circumference. Model 3 was adjusted for the variables in model 2 and additional confounders, including diabetes, CVD, hypertension, hyperlipidemia, asthma, and stroke. Furthermore, the heterogeneity between SII and COPD in the distinct populations was assessed through subgroup analysis for the following categorical variables: age groups, PIR, race, education, diabetes, CVD, hypertension, dyslipidemia, asthma, stroke and smoking status. Restricted cubic spline (RCS) analysis was used to evaluate the non-linear associations between SII and COPD. Finally, in order to assess the robustness of our results, we used propensity score matching (PSM) and matched in a 1:1 ratio. To gain an optimal matching result, a standard caliper of the estimated propensity score was 0.02 and specific inclusion covariates were age, PIR, race, education, Wbc, Mono, Eos, waist-height ratio, waist, diabetes, CVD, smoking status, hypertension, hyperlipidemia, asthma, and stroke. To explore the association between SII and lung function, we applied multivariable linear regression model, including a crude model and an adjusted model with confounding factors selected based on results of univariable linear regression analysis. Then, multivariable linear regression model with RCS was used to test non-linearity. If non-linear association was detected, The threshold effect was calculated using a segmented regression to fit the two-piecewise linear association between Log-SII and lung function. We tested for potential non-linearity based on a likelihood ratio test comparing the linear regression model with the RCS model. To explore the association between SII and severity of COPD, we applied multivariable linear regression model, including a crude model and an adjusted model which adjusted for race, smoking status, education, Wbc count, Mono count, waist-height ratio, waist circumference, and asthma. R statistical software (version 4.2.2) including packages “nhanesR”, “MatchIt”, “pROC”, “tableone”, “lmtest”, and “survey” was used for all statistical analyses and mapping. Alpha was set at < 0.05 for statistical significance, and all analyses were two-sided. A two-sided P value < 0.05 was defined as the significance threshold.

## Results

### Baseline characteristics before matching

In total, 18,349 participants from NHANES (2005 to 2018) were enrolled in the present study, of whom 1,094 (5.96%) had COPD. Among all the participants with COPD, the incidence of COPD was higher in the participants with lower education level and PIR, higher age and Log-SII, but sex did not differ between the two groups in the baseline characteristics (Table 1). The major risk factors for COPD are DM, CVD, hypertension, hyperlipidemia, smoking status, asthma and stroke, the comparison of baseline characteristics showed that participants with the above risk factors were higher than those without risk factors in the prevalence of COPD. Additionally, the optimal cut-off value of Log-SII for COPD was 2.740 (0.643,0.499) using the Youden index, the area under curve (AUC) was 0.5888, 95% CI: 0.571–0.606 (S1 Fig). Simultaneously, individuals were categorized into two groups according to the optimal cut-off value: Q1 group (Log-SII ≤ 2.740) and Q2 group (Log-SII > 2.740). The differences between Q1 group and Q2 group in Wbc count, Hb, Mono count, Eos count, BMI, waist, COPD, DM, CVD, hypertension, smoking status, asthma, stroke, race, education, and sex were statistically highly significant (all *P* < 0.05). Details are described in Table 2.

**Table 1:** Baseline characteristics of all participants before and after matching by COPD.

Variables	Before matching			After matching			
	non-COPD(n = 17255)	COPD (n = 1094)	P value	non-COPD (n = 1041)	COPD (n = 1041	SMD	P value
Age (years)	55.58(0.15)	60.18(0.40)	< 0.0001	60.20(0.43)	59.83(0.41)	0.037	0.52
Sex, n (%)			0.18			0.053	0.37
Female	8802(51.88)	486(48.94)		446(46.77)	468(49.40)		
Male	8453(48.12)	608(51.06)		595(53.23)	573(50.60)		
Age group, n (%)			< 0.0001			0.038	0.50
40–59	9807(65.56)	426(46.74)		403(46.39)	420(48.28)		
60–79	7448(34.44)	668(53.26)		638(53.61)	621(51.72)		
Education, n (%)			< 0.0001			0.070	0.33
college and higher	8970(61.85)	493(52.50)		453(50.54)	478(53.45)		
middle and high school	6234(32.74)	472(40.56)		450(43.29)	446(39.88)		
primary school and less	2051(5.41)	129(6.94)		138(6.16)	117(6.67)		
Race, n (%)			< 0.0001			0.252	< 0.0001
black	3819(10.10)	194(6.76)		281(12.53)	184(6.75)		
mexican	2680(6.59)	57(1.65)		111(4.00)	55(1.67)		
other	3601(11.49)	148(9.80)		163(8.00)	141(9.79)		
white	7155(71.82)	695(81.79)		486(75.47)	661(81.79)		
PIR			< 0.0001			0.021	0.64
< = 1	3215(10.58)	255(16.04)		265(16.18)	235(15.43)		
>1	14040(89.42)	839(83.96)		776(83.82)	806(84.57)		
BMI (kg.m^2^)	29.50(0.09)	29.42(0.29)	0.80	29.57(0.29)	29.47(0.29)	0.014	0.81
Waist-height ratio	0.60(0.00)	0.61(0.00)	0.01	0.61(0.00)	0.61(0.00)	0.002	0.97	
Waist (cm)	101.46(0.22)	103.72(0.67)	0.002	103.75(0.74)	103.68(0.67)	0.004	0.95
Wbc (×10^9^/L)	7.10(0.03)	7.67(0.08)	< 0.0001	7.75(0.10)	7.63(0.08)	0.045	0.33
Hb (g/L)	14.26(0.03)	14.37(0.07)	0.13	14.32(0.06)	14.37(0.07)	0.031	0.60
Mono (×10^9^/L)	0.56(0.00)	0.60(0.01)	< 0.0001	0.62(0.01)	0.60(0.01)	0.083	0.11
Eos (×10^9^/L)	0.20(0.00)	0.24(0.01)	< 0.0001	0.24(0.01)	0.24(0.01)	0.006	0.91
DM			0.003			0.052	0.84
DM	4273(18.65)	329(24.96)		304(22.87)	305(24.09)		
IFG	964(5.98)	60(5.50)		56(6.61)	56(5.50)		
IGT	721(3.83)	45(4.13)		45(3.92)	43(4.03)		
no	11297(71.55)	660(65.42)		636(66.60)	637(66.38)		
CVD, n (%)			< 0.0001			0.001	0.98
no	15165(90.18)	765(73.71)		741(75.50)	753(75.55)		
yes	2090(9.82)	329(26.29)		300(24.50)	288(24.45)		
Hypertension, n (%)			< 0.0001			0.025	0.64
no	8203(52.78)	406(41.86)		392(4346)	391(42.25)		
yes	9052(47.22)	688(58.14)		649(56.54)	650(57.75)		
Hyperlipidemia, n (%)			0.003			0.057	0.26
no	3707(21.19)	203(16.61)		193(19.10)	196(16.91)		
yes	13548(78.81)	891(83.39)		848(80.90)	845(83.09)		
Asthma			< 0.0001			0.012	0.83
no	15324(88.53)	625(58.35)		665(60.16)	625(60.75)		
yes	1931(11.47)	469(41.65)		376(39.84)	416(39.25)		
Stroke			< 0.0001			0.002	0.97
no	16495(96.85)	977(89.73)		917(90.82)	942(90.77)		
yes	760(3.15)	117(10.27)		124(9.18)	99(9.23)		
Smoking status, n (%)			< 0.0001			0.157	0.02
never	9261(54.21)	171(16.90)		200(19.55)	171(17.60)		
former	4759(28.25)	482(44.92)		381(37.83)	468(45.49)		
now	3235(17.53)	441(38.17)		460(42.62)	402(36.91)		
SII	541.37(4.00)	627.09(12.88)	< 0.0001	580.38(17.10)	633.34(13.52)	0.067	0.005
Log-SII	2.68(0.00)	2.73(0.01)	< 0.0001	2.70(0.01)	2.73(0.01)	0.130	0.01

**Notes:** All values represented are weighted means (standard deviation), or counts (weighted percentage).

**Abbreviations:** SD, standard deviation; SMD, standardized mean difference;PIR, poverty index ratio; BMI, body mass index; Wbc, white blood cells; Mono, monocyte; Eos, eosinophils; Hb, hemoglobin; CKD, chronic kidney disease; CVD, cardiovascular disease; DM, diabetes; IFG, impaired fasting glucose; IGT, impaired glucose tolerance; Log-SII, Logarithm-transformed systemic immune-inflammation index; COPD, chronic obstructive pulmonary disease.

**Table 2:** Baseline characteristics of all participants before and after matching by the optimal cut-off value of Log-SII.

Variables	Before matching			After matching		
	Q1	Q2	P value	Q1	Q2	P value
Age (years)	55.94(0.17)	55.70(0.19)	0.23	59.99(0.39)	60.02(0.39)	0.95
Sex, n (%)			< 0.001			0.52
Female	5785(50.27)	3503(53.95)		512(47.11)	402(49.42)	
Male	5907(49.73)	3154(46.05)		652(52.89)	516(50.58)	
Age group, n (%)			0.64			0.63
40–59	6506(64.63)	3727(64.15)		459(46.81)	364(48.09)	
60–79	5186(35.37)	2930(35.85)		705(53.19)	554(51.91)	
Education, n (%)			0.001			0.15
college and higher	6036(61.94)	3427(60.30)		931(54.30)	392(49.50)	
middle and high school	4213(32.14)	2493(34.86)		481(39.47)	415(43.82)	
primary school and less	1443(5.92)	737(4.84)		144(6.23)	111(6.68)	
Race, n (%)			< 0.0001			< 0.0001
black	3014(12.21)	999(6.29)		332(12.71)	133(5.62)	
mexican	1705(6.42)	1032(6.10)		85(2.66)	81(2.87)	
other	2543(12.31)	1206(9.95)		185(9.51)	119(8.31)	
white	4430(69.06)	3420(77.65)		562(75.12)	585(83.20)	
PIR			0.44			0.64
< = 1	2214(11.07)	1256(10.65)		279(16.23)	221(15.25)	
>1	9478(88.93)	5401(89.35)		885(83.77)	697(84.75)	
BMI (kg.m^2^)	29.14(0.11)	30.05(0.12)	< 0.0001	29.30(0.26)	29.77(0.34)	0.30
Waist-height ratio	0.60(0.00)	0.61(0.00)	< 0.0001	0.61(0.00)	0.62(0.00)	0.11
Waist (cm)	100.78(0.26)	102.88(0.26)	< 0.0001	103.05(0.63)	104.48(0.87)	0.20
Wbc (×10^9^/L)	6.56(0.03)	8.04(0.04)	< 0.0001	7.00(0.08)	8.48(0.09)	< 0.0001
Hb (g/L)	14.30(0.03)	14.23(0.03)	0.03	14.40(0.07)	14.28(0.07)	0.21
Mono (×10^9^/L)	0.54(0.00)	0.59(0.00)	< 0.0001	0.59(0.01)	0.63(0.01)	< 0.001
Eos (×10^9^/L)	0.20(0.00)	0.21(0.00)	< 0.0001	0.24(0.01)	0.25(0.01)	0.14
DM			0.01			0.95
DM	2850(17.97)	1752(20.66)		329(23.46)	280(23.60)	
IFG	651(6.01)	373(5.86)		59(5.65)	53(6.44)	
IGT	474(3.71)	292(4.06)		47(3.93)	41(4.04)	
no	7717(72.30)	4240(69.43)		729(66.96)	544(65.92)	
CVD, n (%)			0.05			0.15
no	10277(89.70)	5653(88.43)		859(76.90)	635(73.91)	
yes	1415(10.30)	1004(11.57)		305(23.10)	283(26.09)	
Hypertension, n (%)			< 0.0001			0.40
no	5620(53.90)	2989(49.36)		436(41.84)	347(43.96)	
yes	6072(46.10)	3668(50.64)		728(58.16)	571(56.04)	
Hyperlipidemia, n (%)			0.10			0.63
no	2573(21.45)	1337(20.08)		225(18.46)	164(17.32)	
yes	9119(78.55)	5320(79.92)		939(81.54)	754(82.68)	
Asthma			< 0.001			0.15
no	10253(87.76)	5696(85.41)		732(62.32)	558(58.30)	
yes	1439(12.42)	961(14.59)		432(37.68)	360(41.70)	
Stroke			0.02			0.03
no	11199(96.76)	6273(95.91)		1049(92.26)	840(89.07)	
yes	493(3.24)	394(4.09)		115(7.74)	108(10.93)	
COPD			< 0.0001			0.003
no	11137(95.17)	6118(92.33)		636(50.74)	405(41.96)	
yes	555(4.83)	539(7.67)		528(49.26)	513(58.04)	
Smoking status, n (%)			< 0.0001			0.01
never	6257(54.13)	3175(48.66)		241(21.68)	130(14.79)	
former	3280(29.02)	1961(29.60)		452(40.22)	397(43.91)	
now	2155(16.85)	1521(21.75)		471(38.10)	391(41.30)	
SII	368.20(1.43)	825.29(4.68)	< 0.0001	362.55(4.58)	900.29(18.10)	< 0.0001
Log-SII	2.54(0.00)	2.90(0.00)	< 0.0001	2.54(0.01)	2.92(0.01)	< 0.0001

**Notes:** All values represented are weighted means (standard deviation), or counts (weighted percentage). The Log-SII was divided to two levels by the optimal cut-off value (Log-SII < 2.740 and Log-SII ≥ 2.740).

**Abbreviations:** SD, standard deviation; PIR, poverty index ratio; BMI, body mass index; Wbc, white blood cells; Mono, monocyte; Eos, eosinophils; Hb, hemoglobin; CKD, chronic kidney disease; CVD, cardiovascular disease; DM, diabetes; IFG, impaired fasting glucose; IGT, impaired glucose tolerance; Log-SII, Logarithm-transformed systemic immune-inflammation index; COPD, chronic obstructive pulmonary disease.

### Baseline characteristics after matching

Table 1 showed baseline characteristics of the non-COPD and COPD groups matched on propensity score. A total of 2082 participants, including 1041 of non-COPD and 1041 of COPD were matched according to propensity score matching ratio (1:1). Each variable was equalized and comparable in disparate groupings after matching (standardized mean difference (SMD) < 0.10). S2 Fig presented the distribution of propensity scores before and after matching, implying the matching result was a well-balanced distribution.

### Relationship between SII and COPD

In weighted univariable analysis before matching, Log-SII, Wbc count, Mono count, Eos count, age, waist, waist-height ratio, DM, CVD, smoking status, hypertension, hyperlipidemia, asthma, stroke, PIR, education, and race were significantly associated with the COPD morbidity. Log-SII was a potent predictive indicator for COPD (OR = 2.95, 95% CI: 2.10 to 4.13, p < 0.0001) in S1 Table.

The results of the multivariable logistic regressions for the relationship between Log-SII and COPD were shown in Tables 3 and 4. In all regression models before matching, the influence of Log-SII on COPD was statistically significant (Table 3). Among them, with each 1 unit increase in Log-SII, the risk of COPD increased by a factor of 76% (OR = 1.76, 95% CI:1.19 to 2.61) in model 3, revealing that the Log-SII was positively correlated with the risk of COPD. Likewise, the Q1 group (< 2.740) was the reference in all models, indicating that participants in the Q2 group (≥2.740) was at a higher risk of COPD in all models. Specifically, subjects in the Q2 group vs. those in the Q1 group demonstrated the risk of COPD increased by a factor of 35% (OR = 1.39, 95% CI: 1.16 to 1.68) in model 3. After matching, we explored further the association between Log-SII and COPD under logistic regression models (Table 4). Likewise, results of these regression models proved that Log-SII was an independent risk factor for COPD.

**Table 3:** Association of Log-SII with COPD before matching, NHANES 2005–2018.

COPD	OR (95%CI); p-Value							
	Crude model		Model 1		Model 2		Model 3	
Continuous	2.95(2.10,4.13)	<0.0001	2.10(1.50,2.93)	<0.0001	2.09(1.42,3.09)	<0.001	1.76(1.19,2.61)	0.01
Q1	1.00 (ref).		1.00 (ref).		1.00 (ref).		1.00 (ref).	
Q2	1.64(1.39,1.93)	<0.0001	1.45(1.22,1.72)	<0.0001	1.44(1.20,1.72)	<0.001	1.39(1.16,1.68)	<0.001

**Notes:** The Log-SII was converted from a continuous variable to a categorical variable. Data are presented as OR (95% CI). Crude model was adjusted no covariates. Model 1 was adjusted for age, PIR, race, smoking status, and education. Model 2 was adjusted for Model 1 + Wbc count, Mono count, Eos count, waist-height ratio, and waist. Model 3 was adjusted for Model 2 + DM, asthma, stroke, CVD, hypertension, and hyperlipidemia.

**Abbreviations:** NHANES: the National Health and Nutrition Examination Survey; PIR, poverty index ratio; BMI, body mass index; Wbc, white blood cells; Mono, monocyte; Eos, eosinophils; Hb, hemoglobin; CKD, chronic kidney disease; CVD, cardiovascular disease; DM, diabetes; IFG, impaired fasting glucose; IGT, impaired glucose tolerance; Log-SII, Logarithm-transformed systemic immune-inflammation index; COPD, chronic obstructive pulmonary disease; OR, odds ratio; CI, confidence interval.

**Table 4:** Association of Log-SII with COPD after matching, NHANES 2005–2018.

COPD	OR (95%CI); p-Value							
	Crude model		Model 1		Model 2		Model 3	
Continuous	1.70(1.11,2.61)	0.02	1.70(1.11,2.60)	0.02	1.92(1.18,3.10)	0.01	2.00(1.23,3.24)	0.01
Q1	1.00 (ref).		1.00 (ref).		1.00 (ref).		1.00 (ref).	
Q2	1.42(1.13,1.79)	0.003	1.44(1.13,1.82)	0.003	1.51(1.17,1.96)	0.002	1.54(1.19,1.99)	0.001

**Notes:** The Log-SII was converted from a continuous variable to a categorical variable. Data are presented as OR (95% CI). Crude model was adjusted no covariates. Model 1 was adjusted for age, PIR, race, smoking status, and education. Model 2 was adjusted for Model 1 + Wbc count, Mono count, Eos count, waist-height ratio, and waist. Model 3 was adjusted for Model 2 + DM, asthma, stroke, CVD, hypertension, and hyperlipidemia.

**Abbreviations:** NHANES: the National Health and Nutrition Examination Survey; PIR, poverty index ratio; BMI, body mass index; Wbc, white blood cells; Mono, monocyte; Eos, eosinophils; Hb, hemoglobin; CKD, chronic kidney disease; CVD, cardiovascular disease; DM, diabetes; IFG, impaired fasting glucose; IGT, impaired glucose tolerance; Log-SII, Logarithm-transformed systemic immune-inflammation index; COPD, chronic obstructive pulmonary disease; OR, odds ratio; CI, confidence interval.

### Subgroup analysis

**Fig 2:**
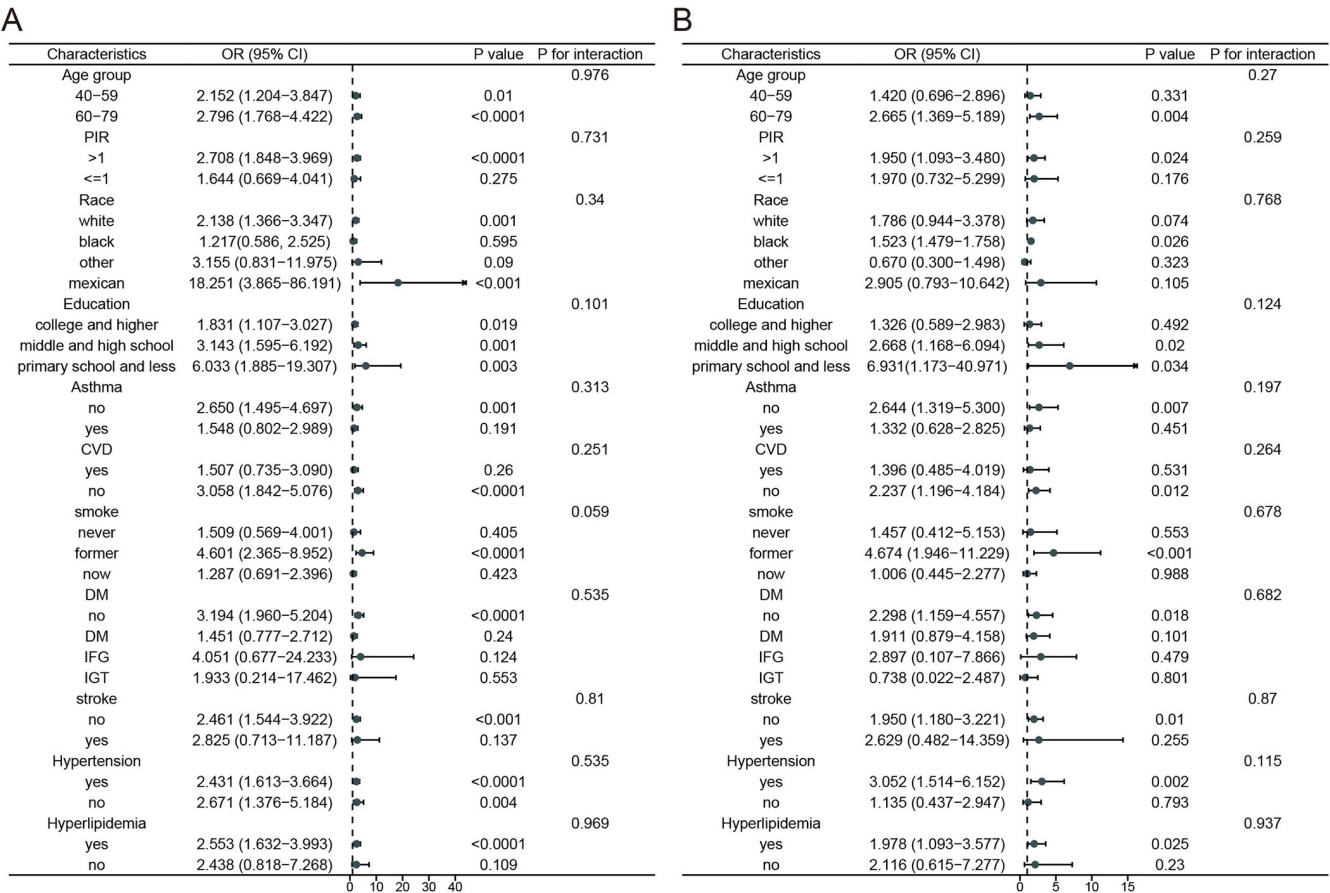
Subgroup analyses of the association between Log-SII and COPD before and after matching, NHANES 2005–2018. **Notes:** Data are presented as OR (95% CI). Adjusted for age, PIR, race, education, Wbc count, Mono count, Eos count, waist, waist-height ratio, DM, asthma, stroke, CVD, smoking status, hypertension, and hyperlipidemia. (A) Subgroup analysis of the association between Log-SII and COPD before matching among all the participants; (B) Subgroup analysis of the association between Log-SII and COPD after matching among all the participants. **Abbreviations:** NHANES, the National Health and Nutrition Examination Survey; PIR, poverty index ratio; BMI, body mass index; Wbc, white blood cells; Mono, monocyte; Eos, eosinophils; Hb, hemoglobin; CKD, chronic kidney disease; CVD, cardiovascular disease; DM, diabetes; IFG, impaired fasting glucose; IGT, impaired glucose tolerance; Log-SII, Logarithm-transformed systemic immune-inflammation index; COPD, chronic obstructive pulmonary disease; OR, odds ratio; CI, confidence interval.

To investigate further the relationship between Log-SII and COPD among different populations, we carried out subgroup analysis before and after matching, stratified by PIR, age group, race, education, DM, CVD, asthma, hyperlipidemia, hypertension, stroke and smoking status. Significant relationships between Log-SII and COPD were shown in all age groups, education, and hypertension stratification variables (*P* < 0.05). But the result of the interaction analysis showed no significant interaction between Log-SII and these variables (all *P* for interaction > 0.05, Fig 2A). Similarly, the matching groups and the non-matching groups showed consistent conclusions in the interaction analysis (Fig 2B).

### RCS analysis

**Fig 3:**
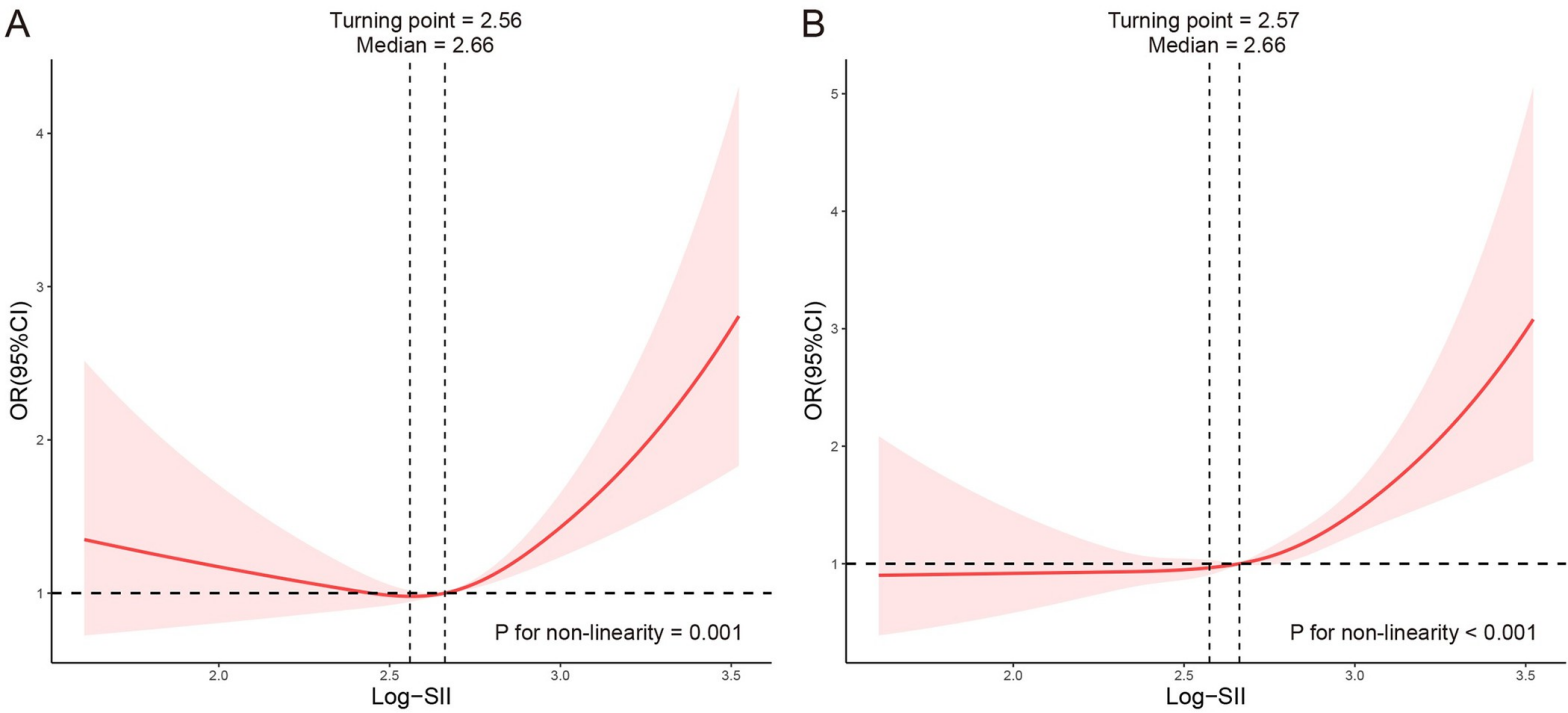
RCS analysis of the association between Log-SII and COPD before and after matching, NHANES 2005–2018. **Notes:** The association was adjusted for age, PIR, race, education, Wbc count, Mono count, Eos count, waist, waist-height ratio, DM, asthma, stroke, CVD, smoking status, hypertension, and hyperlipidemia. The median Log-SII was chosen as a reference. (A) RCS curve of the association between Log-SII and COPD before matching; (B) RCS curve of the association between Log-SII and COPD after matching. **Abbreviations:** RCS, restricted cubic spline; NHANES, the National Health and Nutrition Examination Survey; PIR, poverty index ratio; BMI, body mass index; Wbc, white blood cells; Mono, monocyte; Eos, eosinophils; Hb, hemoglobin; CKD, chronic kidney disease; CVD, cardiovascular disease; DM, diabetes; IFG, impaired fasting glucose; IGT, impaired glucose tolerance; Log-SII, Logarithm-transformed systemic immune-inflammation index; COPD, chronic obstructive pulmonary disease; OR, odds ratio; CI, confidence interval.

The associations between Log-SII and COPD were evaluated with the RCS curves based on the multivariable logistic regression model (Model 3). First, non-matching groups revealed a reversed L-shaped curves relationship (*P* for nonlinear = 0.001) between Log-SII and the risk of COPD (Fig 3A). The turning point appeared around Log-SII of 2.56, and the median number was 2.66. Firstly, the risk of COPD being early stages showed slow changes near the OR of 1, while the latter stages of the Log-SII level, after it reached the turning point, its risk showed a steep rise. Besides, we further had the RCS analysis in matching groups among the association of Log-SII and COPD (Fig 3B). Results revealed the similar reversed L-shaped relationships (*P* for nonlinear < 0.001); Like the relationships between the Log-SII and COPD before matching, the turning point appeared around Log-SII of 2.57, the median number was 2.66.

### Relationship between lung function, COPD severity and SII

**Fig 4:**
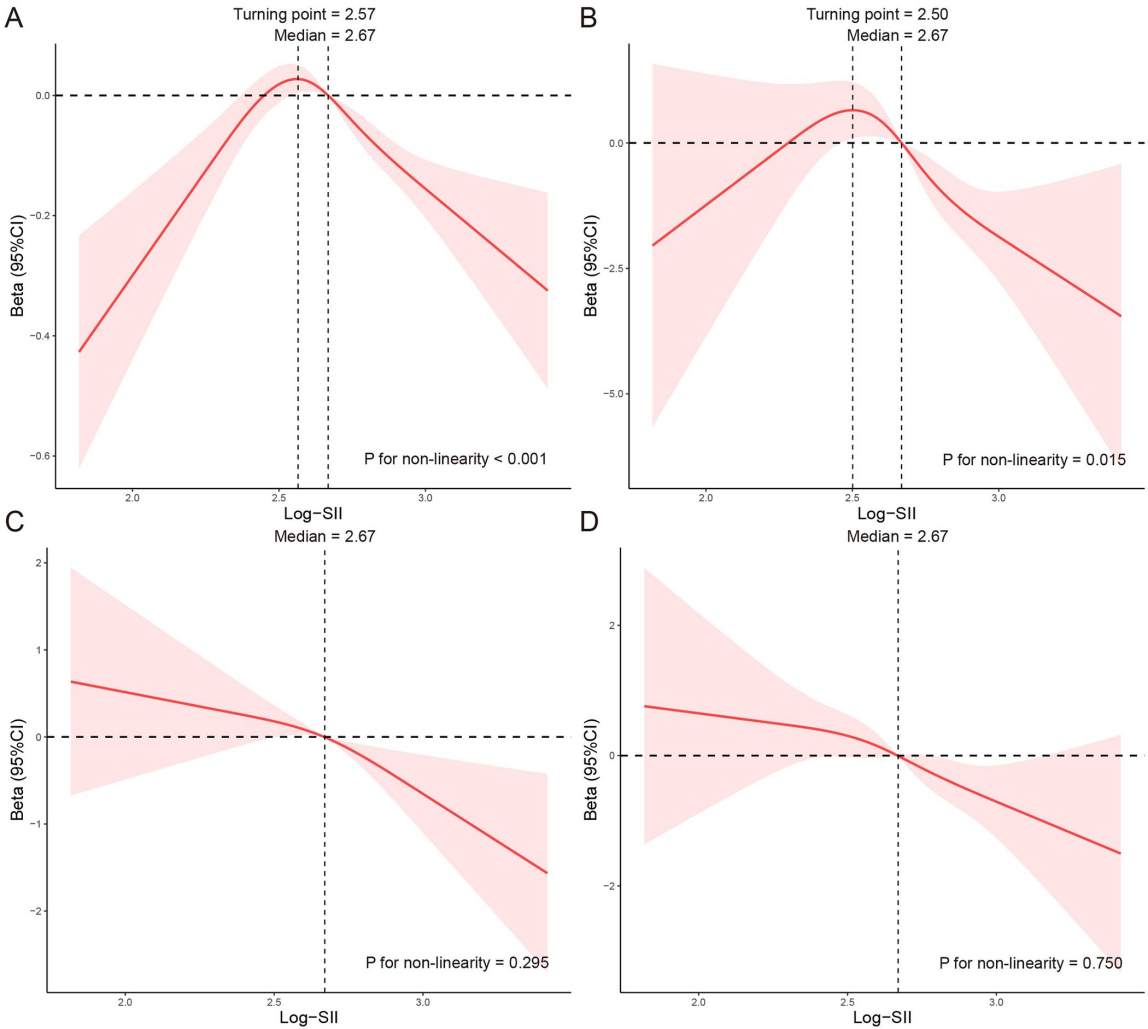
RCS analysis of the association between Log-SII and lung function, NHANES 2007–2012. **Notes:** Adjusted model of FEV1 was adjusted for sex, age, PIR, race, smoking status, education, Wbc count, Hb, BMI, waist-height ratio, DM, asthma, stroke, CVD, hypertension, and hyperlipidemia; Adjusted model of FEV1/FVC% was adjusted for sex, age, race, smoking status, education, Wbc count, Mono count, Eos count, Hb, BMI, waist-height ratio, waist, asthma, stroke, CVD, and hypertension. Adjusted model of FEV1% predicted was adjusted for sex, age, PIR, race, smoking status, education, Wbc count, Mono count, Eos count, Hb, BMI, waist-height ratio, waist, DM, asthma, stroke, CVD, and hypertension; Adjusted model of FEV1/FVC % predicted was adjusted for age, race, smoking status, education, Wbc count, Mono count, Eos count, Hb, BMI, waist-height ratio, waist, DM, asthma, and CVD. The median and turning point of Log-SII was chosen as a reference. (A) RCS curve of the association between Log-SII and FEV1; (B) RCS curve of the association between Log-SII and FEV1% predicted; (C) RCS curve of the association between Log-SII and FEV1/FVC%; (D) RCS curve of the association between Log-SII and FEV1/FVC% predicted. **Abbreviations:** RCS, restricted cubic spline; NHANES: the National Health and Nutrition Examination Survey; PIR, poverty index ratio; BMI, body mass index; Wbc, white blood cells; Mono, monocyte; Eos, eosinophils; Hb, hemoglobin; CKD, chronic kidney disease; CVD, cardiovascular disease; DM, diabetes; FEV1, forced expiratory volume in one second; FVC, forced vital capacity; Log-SII, Logarithm-transformed systemic immune-inflammation index; CI, confidence interval.

The baseline characteristics of lung function and COPD severity are defined in S2 Table. The weighted univariable analysis of lung function and COPD severity are defined in S3 Table. The linear associations of Log-SII with lung function were summarized in Table 5. We found that Log-SII was negatively associated with FEV1, FEV1/FVC, FEV1% predicted and FEV1/FVC% predicted in the unadjusted and adjusted models. Log-SII was not statistically associated with FVC and FVC% predicted in the crude and adjusted model. Then, we conducted RCS curves based on the multivariable logistic regression model to test non-linearity. The adjusted model showed a non-linear association of Log-SII with FEV1 and FEV1% predicted (P for non-linearity were<0.001 and 0.015, respectively). Log-SII had a linear relationship with FEV1/FVC and FEV1/FVC% predicted (P for non-linearity were 0.295 and 0.750, respectively) (Fig 4).

**Table 5:** Association of Log-SII with lung function, NHANES 2007–2012.

Lung function	Crude model		Adjusted model	
	Beta (95%CI)	*P*	Beta (95%CI)	*P*
FEV1	-0.16(-0.25,-0.06)	0.002	-0.10(-0.19,-0.01)	0.04
FVC	-0.12(-0.27,0.03)	0.13	0.26(-0.25,0.57)	0.10
FEV1/FVC %	-1.49(-2.47,-0.50)	0.004	-1.42(-2.48,-0.37)	0.01
FEV1% predicted	-3.65(-6.14,-1.16)	0.005	-3.31(-5.73,-0.89)	0.01
FVC% predicted	-1.77(-3.79,0.25)	0.08	2.81(-7.19,9.42)	0.39
FEV1/FVC% predicted	-2.42(-3.71,-1.14)	<0.001	-1.75(-3.12,-0.37)	0.01

**Notes:** Data are presented as beta (95% CI). Crude model was adjusted no covariates. Adjusted model of FEV1 was adjusted for sex, age, PIR, race, smoking status, education, Wbc count, Hb, BMI, waist-height ratio, DM, asthma, stroke, CVD, hypertension, and hyperlipidemia; Adjusted model of FVC was adjusted for sex, age, PIR, race, education, Wbc count, Hb, BMI, waist-height ratio, DM, asthma, stroke, CVD, hypertension, and hyperlipidemia; Adjusted model of FEV1/FVC% was adjusted for sex, age, race, smoking status, education, Wbc count, Mono count, Eos count, Hb, BMI, waist-height ratio, waist, asthma, stroke, CVD, and hypertension. Adjusted model of FEV1% predicted was adjusted for sex, age, PIR, race, smoking status, education, Wbc count, Mono count, Eos count, Hb, BMI, waist-height ratio, waist, DM, asthma, stroke, CVD, and hypertension; Adjusted model of FVC% predicted was adjusted for sex, PIR, race, smoking status, education, Wbc count, Mono count, Eos count, BMI, waist-height ratio, waist, DM, asthma, stroke, CVD, hypertension, and hyperlipidemia; Adjusted model of FEV1/FVC % predicted was adjusted for age, race, smoking status, education, Wbc count, Mono count, Eos count, Hb, BMI, waist-height ratio, waist, DM, asthma, and CVD.

**Abbreviations:** NHANES: the National Health and Nutrition Examination Survey; PIR, poverty index ratio; BMI, body mass index; Wbc, white blood cells; Mono, monocyte; Eos, eosinophils; Hb, hemoglobin; CKD, chronic kidney disease; CVD, cardiovascular disease; DM, diabetes; FEV1, forced expiratory volume in one second; FVC, forced vital capacity; Log-SII, Logarithm-transformed systemic immune-inflammation index; CI, confidence interval.

We further conducted a threshold effect analysis of Log-SII on FEV1 and FEV1% predicted, and detected the turning point for Log-SII were 2.57 and 2.50 respectively. When the Log-SII was<2.57, the adjusted change in FEV1 per 1 unit Log-SII was 0.44 (95% CI: 0.14 to 0.75); When the Log-SII was≥2.57, the adjusted change in FEV1 per 1 unit Log-SII was -0.33 (95% CI: -0.41 to -0.26). However, when Log-SII was≥2.50, per 1 unit increase was significantly associated with lower FEV1% predicted (beta = −4.35, 95% CI: −7.72 to −0.97). In addition, no significant association was found between Log-SII and FEV1% predicted when the Log-SII was<2.50 (Table 6).

**Table 6:** Association of Log-SII with FEV1 and FEV1% predicted using segmented linear regression.

Log-SII	Crude model		Adjusted Model	
	Beta (95%CI)	P	Beta (95%CI)	P
FEV1				
<2.57	0.65 (0.26,1.05)	0.002	0.44 (0.14,0.75)	0.01
≥2.57	-0.42 (-0.63,-0.21)	<0.001	-0.33 (-0.41,-0.26)	0.02
Likelihood ratio test		<0.001		<0.001
FEV1% predicted				
<2.50	4.16 (-4.10,12.42)	0.32	2.24 (-5.52,10.01)	0.56
≥2.50	-7.16 (-10.83,-3.49)	<0.001	-4.35 (-7.72,-0.97)	0.01
Likelihood ratio test		<0.001		<0.001

**Notes:** Data are presented as beta (95% CI). Crude model was adjusted no covariates. Adjusted model of FEV1 was adjusted for sex, age, PIR, race, smoking status, education, Wbc count, Hb, BMI, waist-height ratio, DM, asthma, stroke, CVD, hypertension, and hyperlipidemia; Adjusted model of FEV1% predicted was adjusted for sex, age, PIR, race, smoking status, education, Wbc count, Mono count, Eos count, Hb, BMI, waist-height ratio, waist, DM, asthma, stroke, CVD, and hypertension.

**Abbreviations:** NHANES: the National Health and Nutrition Examination Survey; PIR, poverty index ratio; BMI, body mass index; Wbc, white blood cells; Mono, monocyte; Eos, eosinophils; Hb, hemoglobin; CKD, chronic kidney disease; CVD, cardiovascular disease; DM, diabetes; FEV1, forced expiratory volume in one second; Log-SII, Logarithm-transformed systemic immune-inflammation index; CI, confidence interval.

The association of severity of COPD with Log-SII was summarized in Table 7. In the crude model, an increase in FEV1% predicted was associated with a significant decrease in Log-SII (beta = -0.18, 95% CI: -0.35 to -0.05, P = 0.02). However, in the adjusted model, the association was no longer statistically significant (beta = -0.09, 95% CI: -0.26 to 0.08, P = 0.27). As for GOLD 1 and GOLD 3, whether atomistic or coarse-grained, an increase in FEV1% predicted was significantly associated with a reduction in Log-SII. But, GOLD 2 did not have statistical significance.

**Table 7:** Association of Log-SII with severity of COPD, NHANES 2007–2012.

Log-SII	Crude model		Adjusted Model	
	Beta (95%CI)	*P*	Beta (95%CI)	*P*
FEV1% predicted				
Continuous	-0.18 (-0.35,-0.05)	0.02	-0.09 (-0.26,0.08)	0.27
GOLD grade				
GOLD 1	-0.33 (-0.55,-0.11)	0.004	-0.23 (-0.45,-0.01)	0.04
GOLD 2	-0.14 (-0.34,0.06)	0.16	-0.07 (-0.27,0.13)	0.46
GOLD 3	-0.51 (-0.89,-0.14)	0.01	-0.34 (-0.67,-0.02)	0.04

**Notes:** Data are presented as beta (95% CI). Crude model was adjusted no covariates. Adjusted model was adjusted for race, smoking status, education, Wbc count, Mono count, waist-height ratio, waist circumference, and asthma.

**Abbreviations:** NHANES: the National Health and Nutrition Examination Survey; Wbc, white blood cells; Mono, monocyte; Eos, eosinophils; FEV1, forced expiratory volume in one second; GOLD, global initiative for chronic obstructive lung disease; Log-SII, Logarithm-transformed systemic immune-inflammation index; CI, confidence interval.

## Discussion

Our large-scale cross-sectional study reported for the association of SII with COPD, lung function and COPD severity based on NHANES database. In this cross-sectional study of 18,349 adults, we observed reversed L-shaped curves relationship between Log-SII and COPD, indicating that after the inflection point, higher SII was associated with an increased risk of COPD. The association between the SII and COPD was still stable after adjustment for confounding factors. The *P* for interaction was not statistically significant after adjusting the confounding factors, showing that the results of different stratification factor subgroups are consistent and reliable. Moreover, In studies of lung function and COPD severity, we found a statistically negative association between the FEV1/FVC%, FEV1/FVC% predicted and SII. Simultaneously, the non-linear, reversed U-shaped curves characterized the relationship between FEV1, FEV1% predicted, and SII. It is worth noting that the increasing of SII have been found to be positively correlated with severity of COPD, particularly manifesting in participants classified under GOLD 1 and GOLD 3.

Numerous studies have linked inflammation and immune stress to the risk and progression of COPD [16, 17]. Several reviews on the pathophysiology of COPD pointed out that inflammation is the basis of the pathological features of COPD. The release of chemical factors stimulated the body to produce an inflammatory response, thus activating an acquired immune response and contributing to the repair and barrier of damaged lung tissue. However, an imbalance between inflammation, immunity, and tissue destruction and repair, as well as persistent local and systemic inflammation, could lead to repeated structural destruction and repair of the lungs, resulting in COPD [18, 19].

Neutrophils, lymphocytes and platelets have previously been found to be associated with the development of COPD patients. Neutrophil is the key inflammatory cells in the pathogenesis of COPD. Many cytokines and proteases secreted by neutrophils are related to lung injury and remodeling in the pathogenesis of COPD. IL-1 and CXCL8/IL-8, neutrophil elastase (NE), matrix metalloproteinase (MMP), myeloperoxidase (MPO), neutrophil extracellular traps (NETs) and high mobility group box 1 (HMGB1) are all shown to be associated with the severity and frequency of COPD [20–23]. Lymphocytes, as inflammatory mediators regulating or protecting function, mediated adaptive immunity and play a vital role in airway remodeling in COPD. Previous studies have focused on the infiltration of T cells, B cells, type 17 helper T cells (Th17) and the reduction of regulatory T cells in the airway [24–28]. Platelet is an atypical inflammatory biomarker, which can regulate innate and adaptive immunity [29, 30]. In a comparative study, platelet activation increased further in COPD patients during acute exacerbation [31]. Shakti Dhar Shukla et al. indicated that the expression of PAFr was up-regulated in the small airways and alveolar epithelium of COPD patients [32].

In the clinical diagnosis and treatment of COPD, neutrophil-to-lymphocyte ratio (NLR), platelet-to-lymphocyte ratio (PLR), blood eosinophil (B-Eos) and lymphocyte-to-high density lipoprotein ratio (LHR) have been reported as new inflammatory markers of COPD. Previous studies have shown that NLR, as a simple and inexpensive inflammatory index, a high value of NLR usually reflected exacerbation of inflammatory state, which is related to COPD complicated with pulmonary hypertension and COPD mortality [33, 34]. At the same time, it was also expected to be a predictor for poor prognosis and mortality of acute exacerbation of chronic obstructive pulmonary disease (AECOPD) [35–37]. PLR is elevated in stable and exacerbated COPD patients, predicting in-hospital mortality [38, 39]. However, there are also some objections. A longitudinal study showed that NLR has no guiding significance in predicting AECOPD [40]. A retrospective analysis also pointed out that the evidence of prognostic value of PLR is still unclear and needs further prospective study and analysis [41]. Blood eosinophil (B-Eos) and lymphocyte-to-high density lipoprotein ratio (LHR) have also been reported as predictors of mortality in COPD patients, but prospective evidence is lacking [41, 42]. So, we can find that different studies have inconsistent diagnostic values for peripheral blood novel inflammatory indicators of COPD. The potential reasons may be related to the different outcome of the study, relatively small sample size or clinical heterogeneity [41].

As a new systemic immune inflammatory marker, SII has been developed for the first time to predict the prognosis of patients with hepatocellular carcinoma after radical resection [6]. Thereafter, it has been widely used as a prognostic indicator in cancer treatment, which has good predictive stability and can objectively represent the balance between inflammatory response and immune response [6–10, 43, 44]. Compared with the more familiar NLR, NLR only considered neutrophils and lymphocytes, but SII incorporated platelets into the equation. This provided a more comprehensive view of the systemic inflammatory response, as platelets played direct roles in both inflammatory and immune response [45]. SII as novel systemic immune inflammatory marker, some studies have investigated the relationship between SII and lung disease. Particularly in lung cancer, SII has the potential to predict poor outcomes for patients with stage III non-small cell lung cancer (NSCLC), and prognosis for patients with surgically resected NSCLC [46, 47]. In addition, several studies have shown that SII is associated with exacerbation in patients with stable COPD, and COPD patients with all-cause mortality [48, 49]. Therefore, we used SII as a comprehensive evaluation index to measure the inflammatory and immune status of individuals, so as to further study its influence on COPD. Our study also showed that SII in peripheral blood is associated with an increased risk of COPD after excluding the confounding factors that led to COPD risk. This observation was consistent with a result reported by Ye et al [49]. In subgroup analysis, the relationship between SII and COPD was not different in special population, and the existence of special population has not been reported in previous studies. RCS analysis showed that the relationship between SII and COPD is an inverse L-shaped curve. It may be worth emphasizing that when Log-SII is less than 2.56, the risk of COPD is almost zero; when Log-SII is greater than 2.56, the risk of COPD rises faster. Simultaneously, the optimum critical value of ROC is 2.740, at which point the corresponding values of SII was 548.87. COPD as one of the common chronic airway inflammatory diseases, significantly reduced quality of life and posed a significant social and economic burden to society. Prevention of COPD, timely diagnosis and early treatment were important factors when it came to improving the prognosis. This is well known that the current gold standard for diagnosis of COPD is pulmonary function test, but there are certain defects, like device inaccuracy, contraindication, or human error defects [50]. Using peripheral blood has advantages over pulmonary function test because it has accessibility, low cost and relative ease of assay standardization [51]. Based on this, we further determined that there was a trend of an inverse L-shaped curve in SII. That is, there was not always a positive relationship, but the COPD risk increased with the increasing of SII after the inflection point. At the same time, the optimal cut-off value of SII for indicating the COPD risk was determined to be 530.09. That is, SII of the subject should be controlled within 530.09 to show a low risk of COPD. Clinically, SII was performed as part of the initial screening evaluation, especially in those patients with chronic respiratory symptoms (such as cough, shortness of breath, etc.) or related risk factors (such as smoking, occupational exposure, etc.), to better help determine the risk of COPD in middle-aged and elderly people. In patients with SII above 530.09, further investigations such as pulmonary function tests, chest imaging, and arterial blood gas analysis may be necessary to diagnose patients with COPD and to assess the severity of COPD. If the diagnosis is confirmed, personalized treatment plan should be formulated based on the patient’s SII. Therefore, SII could be used as part of the screening, diagnosis, and management of COPD patients, but its diagnosis and treatment should be comprehensive, combining a variety of clinical information and examination results to make judgments and decisions.

The possible underlying pathological and physiological mechanisms underlying SII and COPD may be closely related to inflammatory activity, immune dysfunction and systemic inflammatory state. First, there is chronic inflammation in the airways and lung parenchyma, and increased neutrophil and platelet may reflect the extent of the inflammatory response [52–54]. Neutrophil play a key role in acute inflammatory responses, and become particularly active during acute exacerbations of COPD (AECOPD), participating in pathogen clearance and releasing inflammatory mediators [55, 56]. Second, the immune system of COPD patients is in a state of dysregulation, with possible abnormalities in both the number and functionality of lymphocytes, contributing to elevated SII values [17]. Specifically, changes in the number and functions of CD4+ T helper cells and regulatory T cells (Tregs) are associated with the pathological progression and acute exacerbations in COPD [57, 58]. Third, COPD is not only limited to the lung, but also involves systemic inflammatory responses and oxidative stress [59]. The elevated SII represents this systemic effect, including increased risks for cardiovascular complications and other comorbidities [60].

Lung function is often a comprehensive manifestation of lung health, especially in COPD. The change of lung function is important basis for evaluating disease severity, diagnosing, monitoring disease progression and guiding treatment. Therefore, we further studied the association between SII and lung function. In the crude model and the adjusted model, we found that Log-SII was significantly correlated with the FEV1, FEV1% predicted, FEV1/FVC%, and FEV1/FVC% predicted. RCS analysis showed that Log-SII had inverted U relationships with FEV1 and FEV1% predicted, which indicated that Log-SII had positive correlations with FEV1 and FEV1% predicted before the inflection point; After the inflection point, Log-SII was negatively correlated with FEV1 and FEV1% predicted. FEV1 and FEV1% predicted are quantitative and relatively standardized indicators of an individual’s pulmonary airway patency and ventilation function, respectively. This indicated that SII has an inverted U relationship with pulmonary ventilation function. However, no correlation was found between FVC, FVC% predicted and Log-SII. FVC and FVC% predicted as measure of the maximum volume of air that can be forcibly inhaled after full exhalation. This indicated that the change of SII does not affect the total lung volume of individuals. At the same time, there were significant negative linear correlations between FEV1/FVC%, FEV1/FVC% predicted and Log-SII. FEV1/FVC% and FEV1/FVC% predicted as the actual measured ratio and the ratio after considering individual factors are commonly used indicators to judge airway obstruction, respectively. The higher the Log-SII, the stronger the pulmonary airflow limitation.

This relationship between SII and lung function may be due to chronic inflammation of airway and lung parenchyma, which leads to persistent immune inflammatory response. This inflammatory state can be reflected by inflammatory markers in blood. The increasing of these inflammatory markers indirectly reflect the deterioration of lung function. In addition, the local inflammatory reaction caused by acute or chronic lung infection will lead to the destruction of lung structure, affect gas exchange, and thus reduce lung function [61–63].

We further investigated the relationship between SII and the severity of COPD expressed by FEV1% predicted in participants with FEV1/FVC < 0.7 after bronchodilator inhalation. When FEV1% predicted is a continuous variable, SII is negatively correlated with FEV1% predicted in the crude model. However, this relationship becomes insignificant in the adjusted model. When graded according to GOLD, in the adjusted model, SII was negatively correlated with FEV1% predicted in GOLD 1 and GOLD 3 grade. With the increase of the severity of COPD, such as progressing from mild to moderate, severe or even extremely severe, the inflammatory reaction tends to intensify, and the corresponding SII may be higher [64]. Nevertheless, the quantitative relationship between SII and the severity of COPD might exhibit variability contingent upon distinct methodological approaches employed across studies. Therefore, we need well-designed prospective studies and rigorously conducted randomized controlled trials to verify these associations.

This research had several advantages. First of all, our research filled a critical gap of the relationship between COPD, COPD severity, lung function and SII. In addition, it revealed the dose-effect relationship between COPD, lung function and SII. Secondly, NHANES adopted a stratified and multi-stage sampling design to obtain samples representing the non-institutionalized civilian population in the United States, which made our research results widely applicable and universal. Thirdly, this study evaluated many potential confounding factors, and proved the rationality of the model by using tendency score matching method. In addition, we must acknowledge that there are several limitations. First, even if we controlled the potential confounding factors, we couldn’t rule out the role of some unknown or unmeasured other factors. Second, although we used PSM method to eliminate the influence of confounding factors as much as possible and establish relatively perfect randomized controlled grouping, the nature of our cross-sectional study made it difficult to infer causality. Third, data from questionnaires may be affected by recall bias. Fourth, our current results are limited to the American population, so these findings still need to be confirmed in other populations. Fifth, while we adjusted for some possible confounding factors, there may be additional unmeasured confounding factors, such as duration of disease, dosage and time of drugs. Next, in order to improve the practicability of the application model, it was necessary to further verify the predictive value of SII in COPD through future longitudinal studies and RCT studies.

## Conclusion

In conclusion, this cross-sectional study suggested that SII was positively associated with the prevalence of COPD. Simultaneously, the Log-SII level was a reversed L-shaped relationship with the prevalence of COPD in nationally representative adults of US. PSM analysis further validated our findings. Moreover, we observed a negative significant correlation between FEV1/FVC%, FEV1/FVC% predicted and SII, and the Log-SII level was reversed U-shaped curves relationship with FEV1 and FEV1% predicted. The increasing of SII is positively correlated with severity of COPD, especially at GOLD 1 and GOLD 3. These findings could have important implications in routine clinical practice.

## Supporting information

S1 Fig ROC curves of Log-SII. (TIF)

S2 Fig The distributions of PSM before and after matching. (TIF)

S1 Table Univariable logistic regression models of COPD. (XLSX)

S2 Table Baseline characteristics of lung function and COPD severity. (XLSX)

S3 Table The weighted univariable logistic regression models of lung function and COPD severity. (XLSX)
