# Supplementary figures and images for: The association of systemic immune-inflammation index with lung function, risk of COPD and COPD severity: A population-based study
Source: PLoS One. 2024 Jun 14;19(6):e0303286. doi: 10.1371/journal.pone.0303286 (PMC11178193; doi:10.1371/journal.pone.0303286)

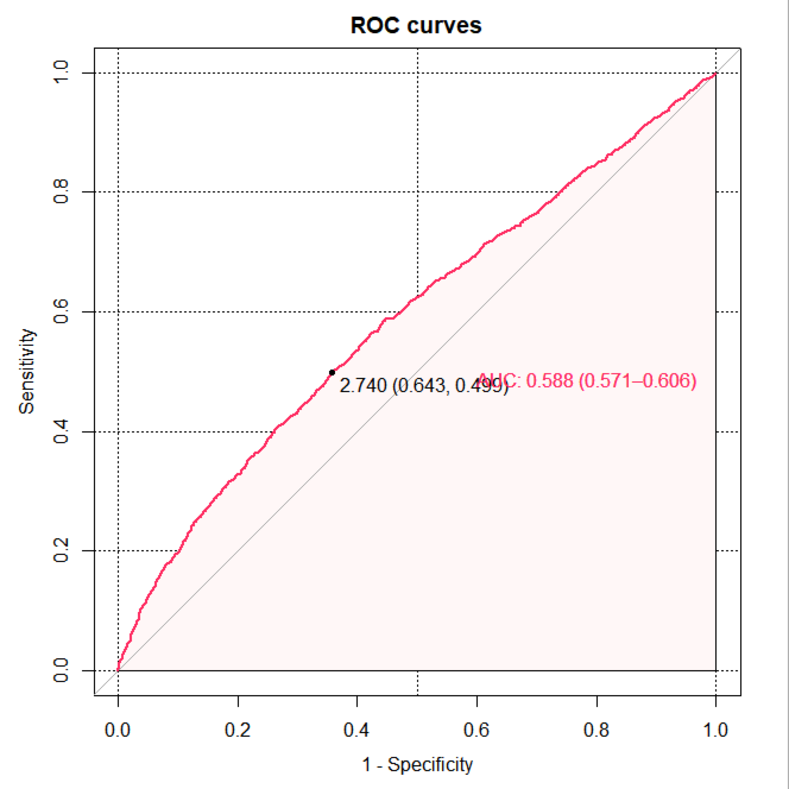

Supplement: S1 Fig — (TIF) [file pone.0303286.s001.tif]

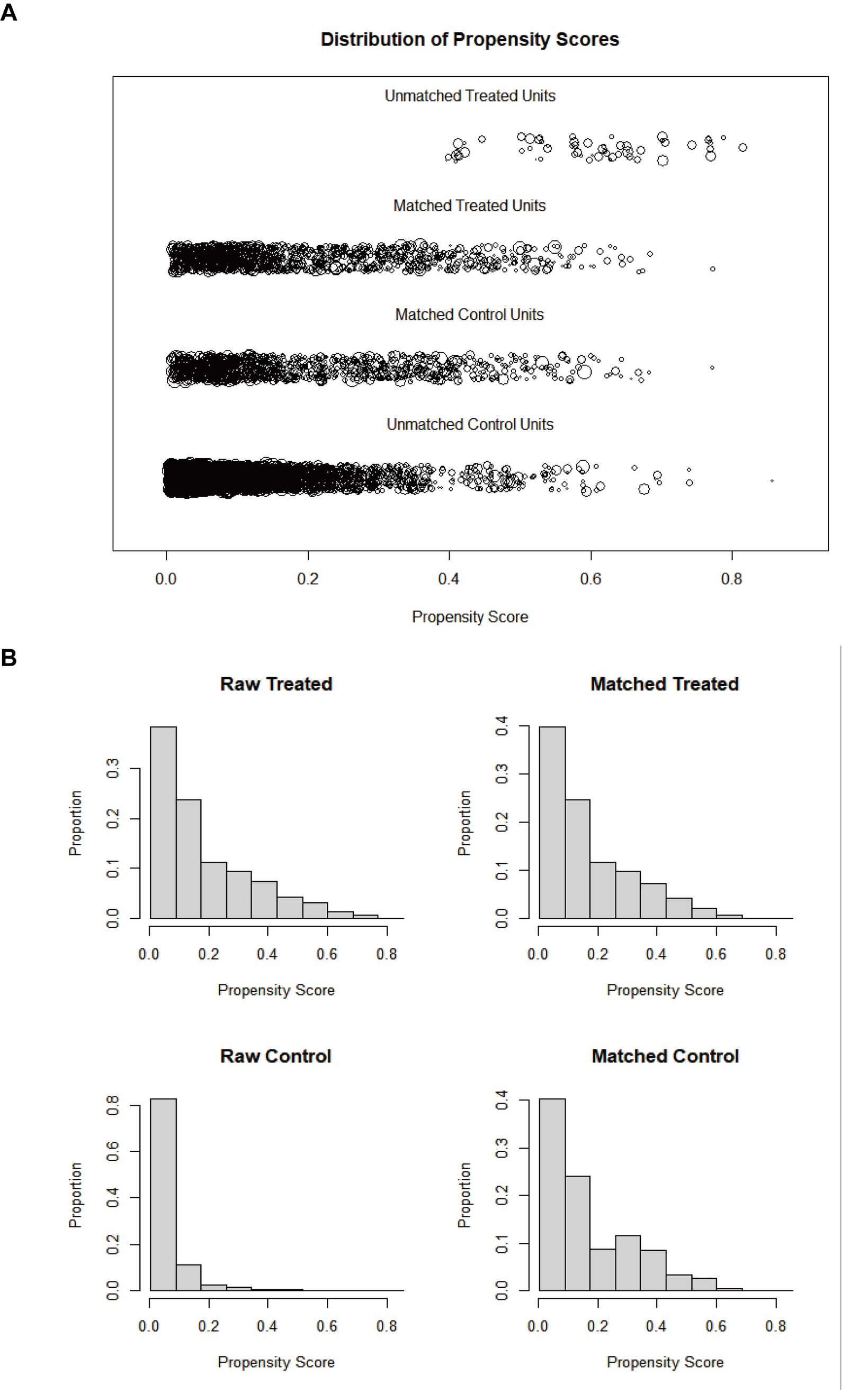

Supplement: S2 Fig — (TIF) [file pone.0303286.s002.tif]
